# Multimodal Sleep Measurement and Alignment Analysis in Outpatients With Major Depressive Episode: Observational Study

**DOI:** 10.2196/82465

**Published:** 2025-12-11

**Authors:** Afrooz Mahir, Nguyen Luong, Ilya Baryshnikov, Annasofia Martikkala, Erkki Isometsä, Talayeh Aledavood

**Affiliations:** 1 Department of Computer Science Aalto University Espoo Finland; 2 Department of Psychiatry University of Helsinki Helsinki Finland; 3 Helsinki and Uusimaa Hospital District Helsinki Finland

**Keywords:** actigraphy, bed sensors, depression, ecological momentary assessment, EMA, sleep monitoring, sleep quantification, smartphone data

## Abstract

**Background:**

Sleep is essential for overall health and plays a critical role in the diagnosis of psychiatric disorders. Although polysomnography remains the gold standard for measuring sleep, its reliance on laboratory settings limits its feasibility for long-term, naturalistic monitoring, particularly for patients with mental disorders.

**Objective:**

This study assesses sleep-tracking reliability and alignment in healthy individuals and patients with mood disorders using wearables, nearables, and ecological momentary assessment, while examining measurement biases and the impact of seasonal and demographic factors on discrepancies across methods.

**Methods:**

We conducted a 14-day study in Finland and enrolled a total of 201 participants, comprising patients with a major depressive episode and healthy controls. Of these, 169 participants with sufficient observations were retained for further analyses. Participants’ sleep patterns (onset, offset, and total sleep time [TST]) were gathered daily from an actigraph (Actiwatch 2), a bed sensor (Murata SCA11H), mobile screen events, and a daily survey. The alignment between sleep measurement methods was evaluated using Bland-Altman plots and Pearson correlation. Linear mixed models were used to assess the effects of demographics, season, and disorder type on the sleep measures alignment.

**Results:**

Patients exhibited greater variability in sleep measures than healthy controls. For sleep onset, mean biases between devices were small and not statistically significant in either group, with moderate to strong correlations. In contrast, sleep offset showed significantly larger biases in patients: actigraph versus bed (+34.9 minutes; *P*=.01), smartphone versus bed (–45.3 minutes; *P*=.004), and actigraph versus smartphone (+78.7 minutes; *P*<.001), while controls exhibited minimal and nonsignificant differences. For TST, smartphone underestimates sleep compared to both bed sensors (–0.71 minutes; *P*<.001) and actigraphs (–1.35 minutes; *P*<.001). Across devices, TST correlations remained low, spanning *r*=0.12 (*P*=.58) to *r*=0.55 (*P*=.10) in controls and *r*=0.17 (*P*=.19) to *r*=0.43 (*P*=.002) in patients. Mixed models showed that older age was linked to better sleep offset alignment between actigraphy and bed sensors (β=–0.02, 95% CI –0.04 to 0.00; *P*=.048), as well as smartphone and bed sensor (β=–0.03, 95% CI –0.06 to 0.00; *P*=.03). Patients with bipolar/borderline personality disorder showed lower TST alignment, and alignment between smartphone and bed sensor was worse in females (β=−1.03, 95% CI −1.74 to −0.33, *P*=.004). Longer daylight duration was also associated with improved alignment in sleep offset and TST.

**Conclusions:**

This study demonstrates the feasibility of using actigraphy, smartphone data, and bed sensors for sleep tracking in naturalistic settings with patients. It highlights measurement biases across devices, the impact of seasonal variations on sleep research in unique geographical regions like Finland, and key demographic factors influencing sleep measurement discrepancies.

## Introduction

Sleep is a fundamental aspect of mental and physical well-being, playing a crucial role in emotional regulation, cognitive function, and overall health [1-3]. Insufficient and excessive sleep are both linked to cognitive impairment, heightened stress, and increased risk of chronic diseases [4-6]. In psychiatric populations, sleep abnormalities are especially pronounced, with insomnia and hypersomnia being core symptoms of major depressive disorder (MDD) [7], and hypersomnia is frequently observed in bipolar disorder (BD) [8]. Moreover, deviations from typical sleep are associated with elevated suicide risk, mood instability, and early relapse indicators in many psychiatric disorders, including mood disorders [9-11]. Given these profound implications, accurately capturing sleep patterns is essential for psychiatric assessments. However, sleep is typically measured in laboratory settings, which limits its applicability for real-world monitoring. This limitation makes obtaining reliable long-term measurements challenging.

Polysomnography (PSG) is the gold standard for sleep assessment but is typically limited to controlled environments [12-14]. Alternative methods, such as sleep diaries and questionnaires, offer greater flexibility but are prone to recall bias [15], often leading to discrepancies between subjective reports and objective measurements [16,17]. Wearable technology, such as actigraphy, provides a more practical solution with reasonable accuracy in tracking some sleep parameters, such as onset and offset [18]. However, actigraphy has limitations, including potential discomfort and the inability to measure physiological markers like heart rate and respiration [19]. More recent advancements in sleep monitoring have introduced contactless methods, such as ballistocardiography-based bed sensors and smartphone-based sleep tracking [20]. While these newer modalities show promise, their validity and comparability, especially in assessing patients with depressive disorders in real-world settings, remain underexplored. To address this gap, we use a multimodal approach, combining actigraphy, bed sensors, smartphone-based tracking, and the ecological momentary assessment (EMA) [21] to assess sleep.

A crucial issue in sleep research is the discrepancy between different measurement modalities. Studies comparing PSG with actigraphy and self-reports have consistently shown variation in onset and offset measurements [13,14]. The divergence is particularly pronounced in psychiatric populations, where sleep misperception—the tendency to perceive one’s sleep inaccurately—is common [15,22]. While actigraphy has been frequently validated against PSG, the role of newer modalities like bed sensors and smartphone data requires further investigation [23].

Beyond measurement discrepancies, demographic, clinical, and environmental factors introduce further variance. For example, chronotype, gender, and age, as well as external variables such as seasons, are associated with sleep patterns [24,25], with women generally reporting longer sleep [26,27]. Using smartphones at bedtime, a behavior more common in younger participants, can decrease agreement between smartphone and actigraphy estimates because actigraphy may register sleep while the smartphone detects ongoing wakefulness [28]. Psychological conditions such as depression further complicate measurement accuracy due to circadian rhythm disruptions [29]. Seasonal fluctuations in daylight and temperature are consistently linked to variation in sleep timing and duration [30], consequently influencing cross-modal alignment. Evidence for these seasonal influences appears across modalities: nearable sensors [31], wearables [24,25,32-34], and smartphone [35,36].

Given these complexities, this study investigates how sleep onset, offset, and total sleep time (TST) measurements compare across actigraphy, smartphone data, bed sensors, and EMA. Additionally, it examines whether these measurements differ between healthy controls and patients with depression, providing insights into the extent of alignment in sleep assessments. Finally, this research explores the demographic, psychological, and environmental factors contributing to discrepancies between measurement modalities, highlighting the sources of error and bias. By addressing these questions, this study contributes to the area of naturalistic sleep research, offering a deeper understanding of sleep monitoring technologies and their implications for psychiatric evaluation, monitoring, and intervention.

## Methods

### Ethical Considerations

The study was approved by the Helsinki and Uusimaa Hospital District Ethics Committee and by Helsinki and Uusimaa Hospital District Psychiatry (approval number §125/2018). Data were anonymized, and participants received four movie tickets as compensation. Participation was voluntary, and individuals were informed that they could withdraw without consequence. All participants received clear information about the study and provided written informed consent.

### Data Collection

The MoMo-Mood study [37] explored the effectiveness of wearable technology in monitoring sleep and mood in individuals with psychiatric disorders as well as finding behavioral markers of depression from passively collected data [37,38]. The study included 164 participants, of which 133 were diagnosed with a current major depressive episode, including 85 patients with MDD, 27 with borderline personality disorder (BPD), and 21 with BD, alongside 31 healthy controls. Additionally, a pilot study was conducted with 37 participants, including 14 patients with MDD and 23 healthy controls. The main and the pilot studies had a similar design and data from both studies were included in the analysis.

Participants were recruited voluntarily from mood disorder outpatient services at Helsinki University Hospital Mood Disorder Division, Turku University Central Hospital Department of Psychiatry, and City of Espoo Mental Health Services, and healthy controls were recruited from the general community. Eligibility required a confirmed diagnosis of MDD, BD, or BPD, and individuals with psychotic symptoms, current substance use disorder, or acute suicide risk were excluded. Healthy controls were screened to ensure the absence of clinical depression. In the pilot study, patients were asked to participate for 6 months and healthy controls for 1 year, while in the MoMo-Mood study participants were invited to take part for up to 1 year. Participation length varied, resulting in differences in the amount of data contributed. Enrollment occurred on an ongoing basis, with participants joining and leaving at different times. Participation was voluntary, and individuals were informed that they could withdraw without consequence. All participants received clear information about the study and provided written informed consent. The study was approved by the Helsinki and Uusimaa Hospital District Ethics Committee and by Helsinki and Uusimaa Hospital District Psychiatry. Data were anonymized, and participants received 4 movie tickets as compensation. Further details on study design, participants recruitment, and data collection procedure can be found in previous studies [37,38].

The study period was divided into two phases: an initial 2-week active phase and a subsequent passive phase lasting up to 1 year. In this work, we focused exclusively on the active phase of data collection. During this phase, participants wore wrist-worn actigraphy devices and used bed sensors to monitor sleep patterns. Concurrently, smartphone and EMA data was gathered through the AWARE app [39], which tracked various behavioral metrics [37]. Participants were also prompted 5 times daily to report on their mood, energy levels, and other psychological states using a 7-point Likert scale. Participants returned the actigraphy devices and bed sensors after the active phase, but smartphone data collection continued during the passive phase. The duration of the active phase was determined based on the amount of time actigraphs could collect data without needing to be recharged. Charging the actigraphs would have significantly increased the burden on participants and was not deemed feasible. A more detailed description of the questions can be found elsewhere [40].

### Sleep and Activity Monitoring Protocol

Participants, were provided with actigraphy devices (Phillips Actiwatch 2, Philips Respironics) to measure activity levels and sleep for 2 weeks. The devices can operate during this period without recharging to ensure that participants did not have to remove the device during the monitoring session. Participants were required to wear the devices on the wrist for as long as they could and were instructed to remove the device only during sauna use, which is a common activity in Finland.

In addition, nearable devices, including bed sensors and smartphones, were also used for sleep monitoring. The Murata SCA11H (Murata Electronics) nodes, based on ballistocardiography technology, were used for bed sensing. Each participant received a preconfigured Wi-Fi router to enable automatic data transfer to the study server. The participants were asked to place the Murata SCA11H node under their mattress, positioned close to but not directly beneath them. Alternatively, they could be attached to the bed frame near the participant. The sensors processed information on site and produced metrics such as pulse rate, heart rhythm variability, breathing rate, cardiac output, and signal intensity at a 1 Hz rate [41].

Smartphones were used for sleep tracking due to their noninvasive nature and ability to monitor behaviors [20]. We collected communication timestamps (calls and texts), anonymous contact identifiers, smartphone screen activity events (screen on, off, lock, and unlock), location data, app usage patterns, and battery state. In this study, we only analyzed screen events to identify sleep periods.

Furthermore, smartphones facilitated the use of EMA, which was used to gather real-time data on sleep and participants’ emotional states throughout the study [37]. During the active phase, participants received multiple EMA prompts daily on their smartphones. These included a morning questionnaire to assess sleep from the previous night and an evening questionnaire to track the activities of the day. Additionally, a total of 3 randomly timed prompts were sent throughout the afternoon within specific time ranges. The morning questionnaire included a single item asking participants to report how long they had slept during the previous night. The item was answered using categorical response options rather than an open text field. The response options were: under 5 hours, 5-6 hours, 6-7 hours, 7-8 hours, 8-9 hours, 9-10 hours, and over 10 hours. Participants were prompted to complete the morning questionnaire at a fixed time in the morning rather than immediately upon awakening, and they were given a specific time window to respond. Similarly, the evening questionnaire was completed within a designated evening window. This schedule allowed for collecting subjective data on sleep, mood, energy levels, physical activity, and other psychological factors [40]. Data from all modalities were gathered through the Niima data collection platform [42] and was preprocessed with the Niimpy behavior analysis toolbox [43].

### Device Data Export Procedures and Analysis

Sleep data were derived using proprietary algorithms from both the actigraph and bed sensor, which were then transformed into sleep parameters for analysis. For the actigraph, raw data were aggregated into 30-second intervals, with each interval assigned 1 of 3 possible labels based on activity levels. The “ACTIVE” label indicated high activity, indicating the user was awake and moving. The “REST” label represented low activity, suggesting the user was resting but not necessarily asleep. The “REST-S” label denoted sustained low activity, indicating that the user was likely asleep. To identify sleep episodes, the data was sorted chronologically, and continuous REST-S intervals were grouped together and treated as a single sleep episode.

The bed sensor collected raw data on heart rate and respiratory rate at 1-second intervals. This dataset was processed using the manufacturer’s algorithm to generate labels indicating bed occupancy and physiological states [37]. Sleep periods were identified based on the status variable, where a status of “1” indicated sleep, and “0,” “2,” and “3” represented nonsleep states (eg, not in bed, awake in bed, or signal overload). Continuous status “1” intervals were treated as sleep episodes and grouped together as part of the same sleep period.

For the smartphone data, sleep was inferred based on the lock and unlock status as indicators of inactivity, following the procedure described by Aledavood et al [37] and used in previous studies [44,45]. Periods of inactivity, identified when the device was locked, were assessed to determine sleep periods. The longest stretch of inactivity was classified as nocturnal sleep. The lock and unlock status was converted into a binary format, and the longest inactivity periods were tracked to calculate sleep onset, sleep offset, and TST.

In addition, the EMA questions and responses, initially in Finnish, were translated into English. Data were selected based on responses to the question, “How many hours did you sleep last night?” to examine the relationship between subjective and sensor-based sleep data. Categorical TST ranges were transformed into numerical values (eg, “6-7 hours” was converted to 6.5 hours).

### Transforming Raw Data to Sleep Measures

Data preprocessing was performed on the smartphone, actigraph, and bed sensor data. First, missing data were removed to ensure completeness. Second, timestamps were standardized to the Europe/Helsinki time zone to maintain consistency across all data sources. Third, sleep data were aligned using a 3 PM to 3 PM time frame, meaning each 24-hour period started and ended at 3 PM each day. This approach was chosen to account for variations in sleep schedules and to standardize sleep cycle measurements across participants.

A 5-minute threshold was applied to identify sleep periods, allowing gaps of up to 5 minutes between consecutive sleep intervals to be treated as part of the same sleep episode. TST outside the 3-13 hour range was excluded, following established guidelines [46]. This threshold was set to exclude implausible TST values, as unusually short or long sleep episodes could indicate naps, data errors, or atypical patterns.

**Figure 1:**
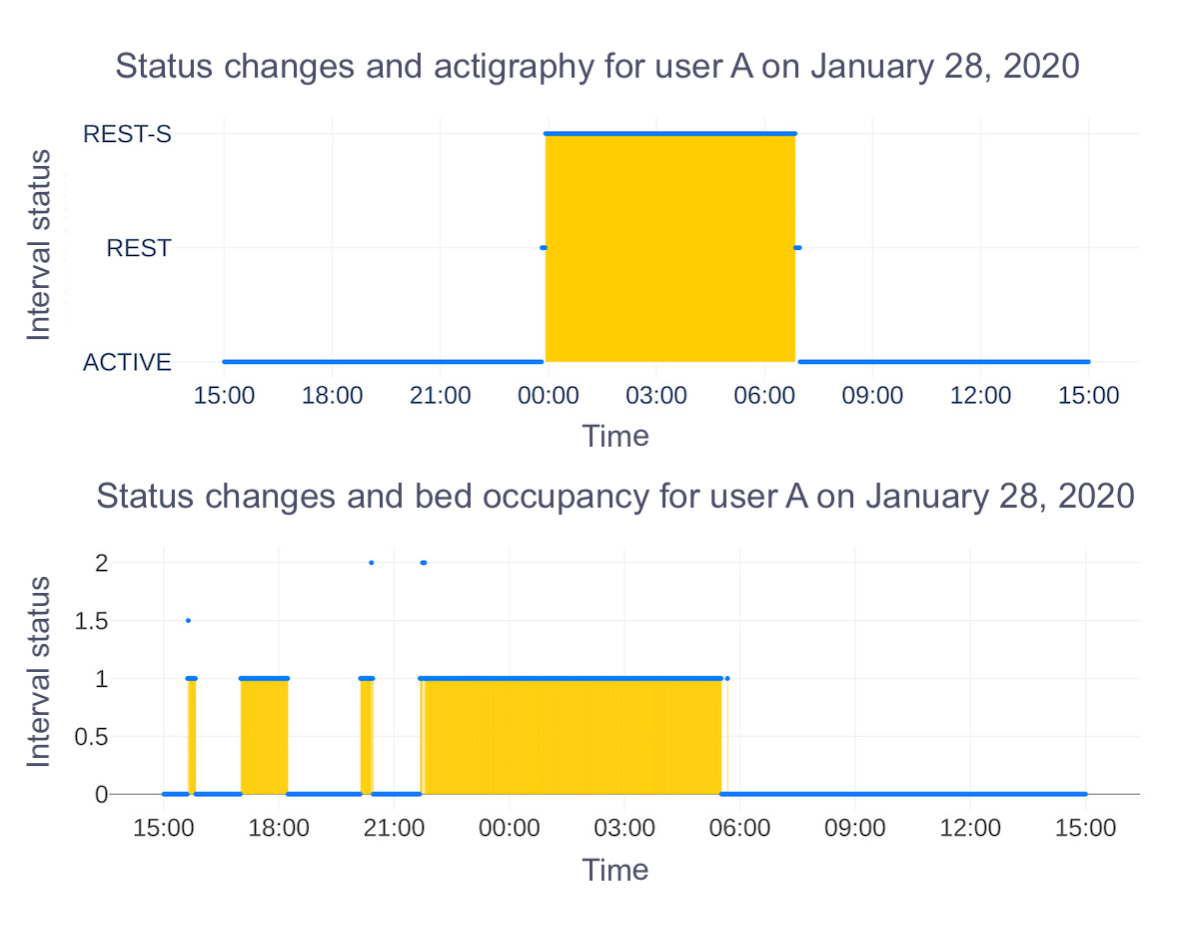
Status changes identified by proprietary algorithms of the actigraph and bed sensor. For the actigraph, the sleep period began at 23:55 and ended at 06:51, marked by “Rest-S,” while the bed sensor indicated “Status 1,” showing the bed was occupied and the user was asleep from 21:40 to 05:30. This displays differences in sleep period detection and wakefulness between the two modalities.

Finally, sleep onset and offset were determined by identifying the start and end times of the longest detected sleep episode within each 24-hour period. Participants with missing values for these parameters were excluded from the analysis. Figure 1 illustrates how sleep periods were identified using the sleep labels provided by the manufacturer for both the actigraph and bed sensor.

### Missing Data and Outlier Handling Procedures

Although 164 participants were initially recruited for the main study, 13 (7.9%) participants did not submit passive data and were removed from all evaluations. Additionally, missing data resulted from various factors, including device noncompliance, such as failing to wear or charge the device, user dropout, inconsistent usage, and data cleaning procedures. Technical issues, such as battery depletion, sensor malfunctions, or connectivity failures, may have also contributed to data loss. After filtering out missing data, the sample included 173 unique participants from both the main and pilot studies. The datasets included 102 actigraph users, 108 bed sensor users, and 148 smartphone users.

Additionally, the 2 largest outliers from both the onset and offset columns were identified and removed to limit the impact of extreme values. Due to the overlap between the outliers in both columns, a total of 3 outliers were removed from the bed sensor and actigraph datasets and 2 from the smartphone dataset, resulting in the exclusion of 8 observations in total. Manual checks revealed that these outliers were due to device-related issues, including improper device wear for the actigraph, placement or configuration errors for the bed sensor, and technical problems with the smartphone data collection. Following data cleaning, the final sample size consisted of 172 participants and was as follows: actigraph (29 healthy controls, 70 patients; n=99), bed sensor (34 healthy controls, 71 patients; n=105), smartphone (46 healthy controls, 100 patients; n=146), and EMA (48 healthy controls, 109 patients; n=157). Furthermore, a total of 3 participants from the pilot study did not provide information on age or gender and, therefore, their data were excluded from further analyses, resulting in a final sample of 169 participants.

### Statistical Analysis

To assess the agreement between different sleep measurement modalities, Bland-Altman plots [47] were created by plotting the differences between paired measurements against their mean values. This approach provides a visual assessment of the differences between two modalities, revealing any systematic bias as well as the limit of agreement (LoA). Sleep onset, offset, and TST comparisons were made across modalities using this approach, with 95% LoA calculated. To account for day-to-day variability within each participant, data were aggregated by averaging individuals’ data across multiple days. Pearson correlation analysis was conducted to quantify the relationships between sleep parameters from different modalities. Normality was first assessed using the Shapiro-Wilk test [48]. If deviations from normality were observed, quantile-quantile plots were used for cross-checking [49]. The size of the correlation coefficients was interpreted based on the thresholds from the guidelines suggested by Mukaka [50]. A high correlation ranged from 0.70 to 1.00, a moderate correlation ranged from 0.50 to 0.70, a low correlation was between 0.30 and 0.50, and a negligible correlation was considered when ranged from 0.00 to 0.30. Finally, 95% CIs were calculated for the regression slopes.

Linear mixed models [51,52] were used to investigate factors associated with the alignment of sleep parameters between modalities and to account for the repeated measurements within each participant. The alignment of sleep parameters between measurement modality pairs was used as the dependent variable, represented as the absolute difference between the two modalities. The models adjusted for demographic factors, including age and gender. Chronotype was included as a fixed effect, measured by the Morning-Eveningness Questionnaire (MEQ), with higher scores indicating morningness preferences and lower scores indicating eveningness preferences. Given the unique geographical position of Finland, the seasonal factor was controlled for using day length duration. Between-group differences were evaluated by comparing each of the 3 patient groups with the healthy control group, which was used as the reference category. Participants were included as the random effect. The model was performed in the R Statistical Software (version 4.3.1; R Core Team) [53] using the *lme4* package [54]. All other analyses were performed in Python (version 3.12, Python Software Foundation) [55].

## Results

### Descriptive Statistics

The demographic characteristics of the participants are outlined in Table 1. Additionally, descriptive statistics for sleep onset, offset, and TST across modalities are presented in Table 2, based on daily averages to account for daily fluctuations. In the control group, sleep onset varies between modalities. The earliest average sleep onset was recorded by the actigraph (11:48 PM, SD 70 minutes), while the smartphone reported the latest (12:01 AM, SD 106 minutes). The bed sensor recorded a mean onset time of 11:58 PM (SD 67 minutes). In the patient group, the mean onset times were generally later, as the bed sensor recorded the earliest average onset at 12:19 AM (SD 100 minutes) and the actigraph reported the latest at 12:35 AM (SD 92 minutes). Lastly, the smartphone recorded a mean onset time of 12:30 AM (SD 101 minutes) for patients.

**Table 1:** Demographic characteristics of participants (N=169).

Characteristic			Value
Age (years), mean (SD)			35.1 (12.8)
Female, n (%)			125 (73.9)
MEQ^a^ score, mean (SD)			39.2 (5.2)
**Group, n (%)**			
	Major depressive disorder	78 (46.2)	
	Control	50 (29.6)	
	Bipolar disorder	21 (12.4)	
	Borderline personality disorder	20 (11.8)	

^a^MEQ: Morning-Eveningness Questionnaire.

**Table 2:** Mean sleep parameters (onset, offset, and total sleep time) and SDs across different assessment methods for control and patient groups.

Parameter	Group	Bed sensor, mean (SD)	Actigraph, mean (SD)	Smartphone, mean (SD)	EMA^a^, mean (SD)
**Onset**					
	Control	23:58 (67 min)	23:48 (70 min)	00:01 (106 min)	—^b^
	Patients	00:19 (100 min)	00:35 (92 min)	00:30 (101 min)	—
**Offset**					
	Control	07:33 (87 min)	07:44 (74 min)	07:06 (72 min)	—
	Patients	08:24 (119 min)	08:58 (91 min)	07:32 (92 min)	—
**Total sleep time (duration)**					
	Control	7 h 35 min (78 min)	7 h 55 min (62 min)	7 h 10 min (87 min)	7 h 14 min (35 min)
	Patients	8 h 5 min (107 min)	8 h 22 min (60 min)	7 h 7 min (78 min)	7 h 26 min (68 min)

^a^EMA: ecological momentary assessment.

^b^Not applicable.

Sleep offset showed a similar pattern, with differences between groups and measurement modalities observed. In the control group, the smartphone reported the earliest mean offset time (7:06 AM, SD 72 minutes), while the actigraph recorded the latest (7:44 AM, SD 74 minutes). The bed sensor estimated a mean offset time of 7:33 AM (SD 87 minutes). In the patient group, offsets occur later, with the smartphone recorded the earliest mean offset time at 7:32 AM (SD 92 min) and the actigraph reported the latest at 8:58 AM (SD 91 min). Finally, the bed sensor reported a mean offset of 8:24 AM (SD 119 min). All modalities indicate a trend of delayed offset times among patients compared to controls. Furthermore, patients show greater variability in sleep offset times, suggesting more inconsistency in their wake-up patterns.

Finally, TST was evaluated using both objective measurements and subjective reports from the EMA questionnaires. In the control group, the smartphone recorded the shortest mean TST (7 hours 10 minutes, SD 87 minutes), while the actigraph recorded the longest (7 hours 55 minutes, SD 62 minutes). The bed sensor estimated a mean TST of 7 hours 35 minutes (SD 78 minutes), and subjective reports from the EMA questionnaires indicated a mean of 7 hours 14 minutes (SD 35 minutes). For the patient group, the mean TST was generally longer, with the smartphone recording the shortest mean TST at 7 hours 7 minutes (SD 78 minutes) and the actigraph recording the longest at 8 hours 22 minutes (SD 60 minutes). The bed sensor estimated a mean TST of 8 hours 5 minutes (SD 107 minutes), and EMA responses showed a mean TST of 7 hours 26 minutes (SD 68 minutes). Overall, the findings showed relatively consistent trends across the modalities in measuring TST.

### Alignment of Sleep Parameters Across Assessment Modalities

#### Sleep Onset

**Figure 2:**
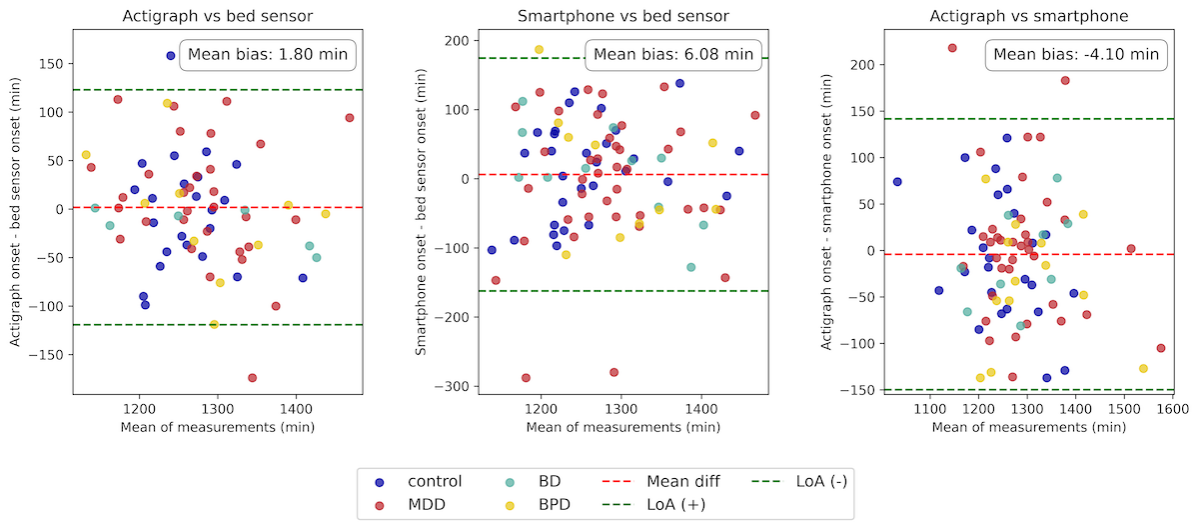
Bland-Altman plot comparing sleep onset times across modalities, with data points color-coded by group. The red dashed line indicates the mean bias, and the green dashed lines represent the 95% limit of agreement (LoA). The smallest mean bias is observed between the actigraph and bed sensor at 1.80 minutes.

**Figure 3:**
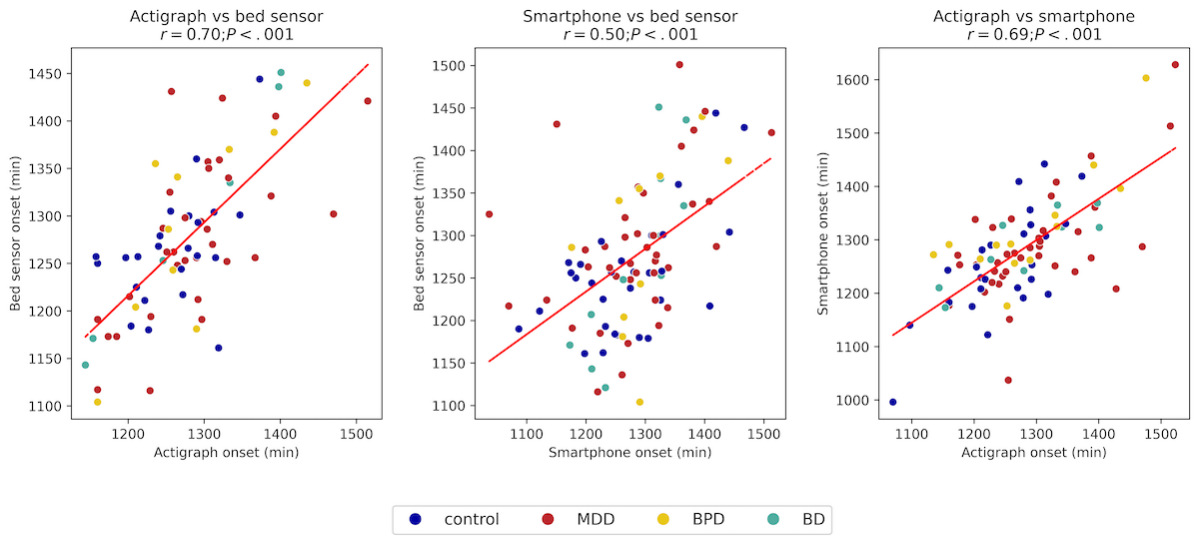
Scatter plots comparing sleep onset times across different modalities, with each point representing a paired dataset. Data points are color-coded to distinguish control and patient groups. All comparisons show positive correlations, with the strongest correlation observed between the actigraph and bed sensor.

Bland-Altman plots comparing sleep onset times across modalities are presented in Figure 2. The mean difference between sleep onset was not statistically significant (Table 3). However, sleep onset showed significant positive correlations across modalities, as displayed in Figure 3. Actigraph and bed sensor onset times had the strongest correlation (*r*=0.70, 95% CI 0.58-0.96; *P<.*001), indicating a high level of agreement between these modalities. The correlation between actigraph and smartphone onset times was moderate (*r*=0.69, 95% CI 0.60-0.95; *P<.*001), indicating that these two modalities capture similar sleep onset patterns. The correlation between smartphone and bed sensor onset times was also moderate (*r*=0.50, 95% CI 0.32-0.69; *P<.*001), suggesting greater variability in smartphone-based measurements.

**Table 3:** Mean bias and statistical comparison of sleep metrics across assessment methods.

Sleep metric and comparison			Mean bias (SD)		*t* test (*df*)	*P* value
**Onset**						
	Actigraph vs bed sensor	1.80 (62.23)		0.24 (70)		.81
	Smartphone vs bed sensor	6.08 (86.42)		0.67 (89)		.51
	Actigraph vs smartphone	–4.10 (74.78)		–0.50 (83)		.62
**Offset**						
	Actigraph vs bed sensor	30.39 (85.26)		3.00 (70)		<.001
	Smartphone vs bed sensor	–37.86 (107.19)		–3.35 (89)		<.001
	Actigraph vs smartphone	64.85 (86.78)		6.85 (83)		<.001
**Total sleep time**						
	Actigraph vs bed sensor	28.57 (103.03)		1.51 (98)		.13
	Actigraph vs smartphone	59.21(87.56)		6.29 (98)		<.001
	Actigraph vs EMA^a^	58.51 (66.42)		5.43 (98)		<.001
	EMA vs bed sensor	–24.9 (93.15)		–2.49 (104)		.01
	EMA vs smartphone	22.61 (79.74)		1.81 (120)		.07
	Smartphone vs bed sensor	–34.55 (111.37)		–3.57 (104)		<.001

^a^EMA: ecological momentary assessment.

#### Sleep Offset

**Figure 4:**
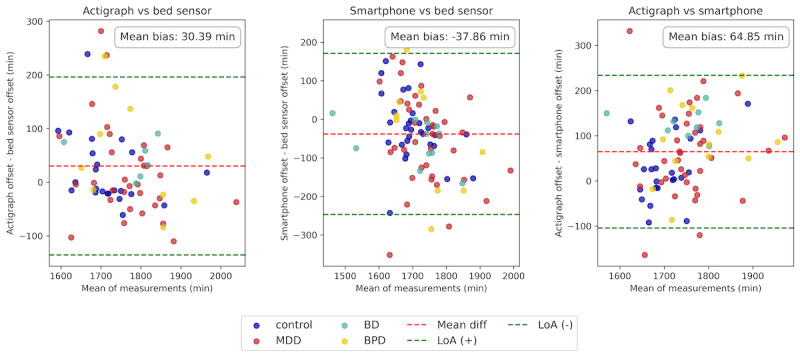
Bland-Altman plot comparing sleep offset times across modalities, with data points color-coded by group. The red dashed line indicates the mean bias, and the green dashed lines represent the 95% limit of agreement (LoA). The actigraph records later offsets, while the smartphone records earlier offsets relative to other modalities. The largest mean bias is observed between the actigraph and smartphone at 64.85 minutes.

Figure 4 presents Bland-Altman plots comparing sleep offset times across the different measurement modalities. The mean bias for sleep offset across all modalities was statistically significant (*P<.*001). In particular, the mean bias between the actigraph and bed sensor offsets was 30.39 (SD 85.26; 95% CI 10.36-50.43) minutes, indicating a positive bias, where the actigraph tends to report later onset times compared to the bed sensor. The smartphone offset differed from the bed sensor by a mean of –37.86 (SD 107.19; 95% CI –60.18 to –15.53) minutes, showing a negative bias, with the smartphone tending to report earlier offset times compared to the bed sensor. Lastly, the mean difference between the actigraph and smartphone offsets was 64.85 (SD 86.78; 95% CI 46.13-83.57) minutes, reflecting a larger positive bias, with the actigraph reporting significantly later onset times compared to the smartphone.

**Figure 5:**
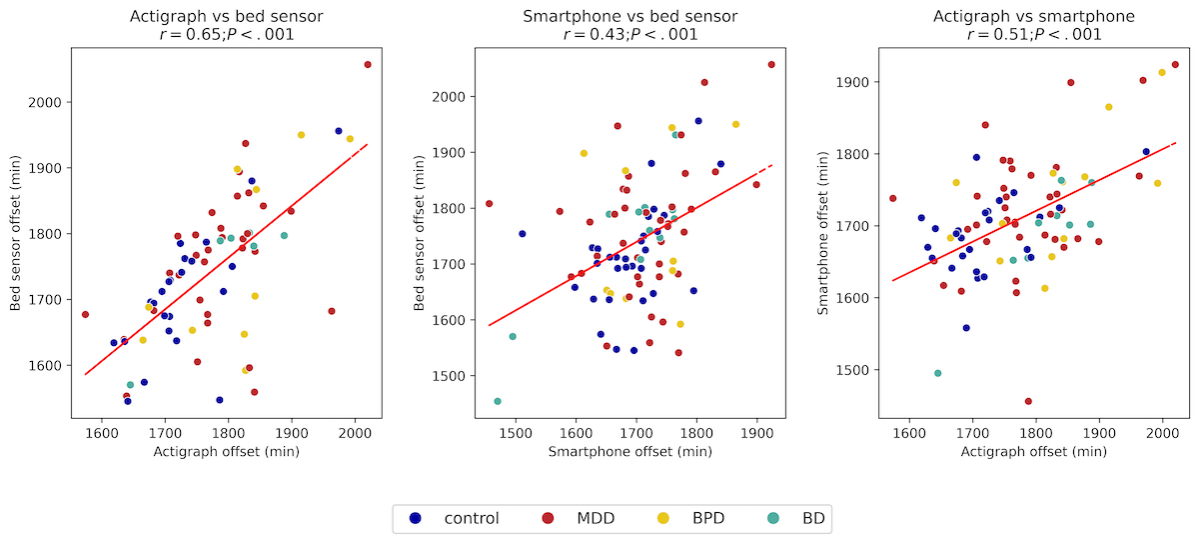
Scatter plots comparing sleep offset times across different modalities. All offsets show a mild positive correlation, with the strongest correlation observed between the actigraph and bed sensor.

Moreover, all modalities showed moderate to low correlation in measuring sleep offset (Figure 5). The highest correlation was observed between the actigraph and bed sensor (*r*=0.65, 95% CI 0.57-1.01; *P<.*001), followed by the correlation between the actigraph and smartphone (*r*=0.51, 95% CI 0.27-0.59; *P<.*001). The smartphone and bed sensor showed the lowest correlation (*r*=0.43, 95% CI 0.34-0.88; *P<.*001). Results indicated moderate to low alignment in sleep offset measurements across modalities, with greater variability observed in smartphone-based measurements.

#### Total Sleep Time

**Figure 6:**
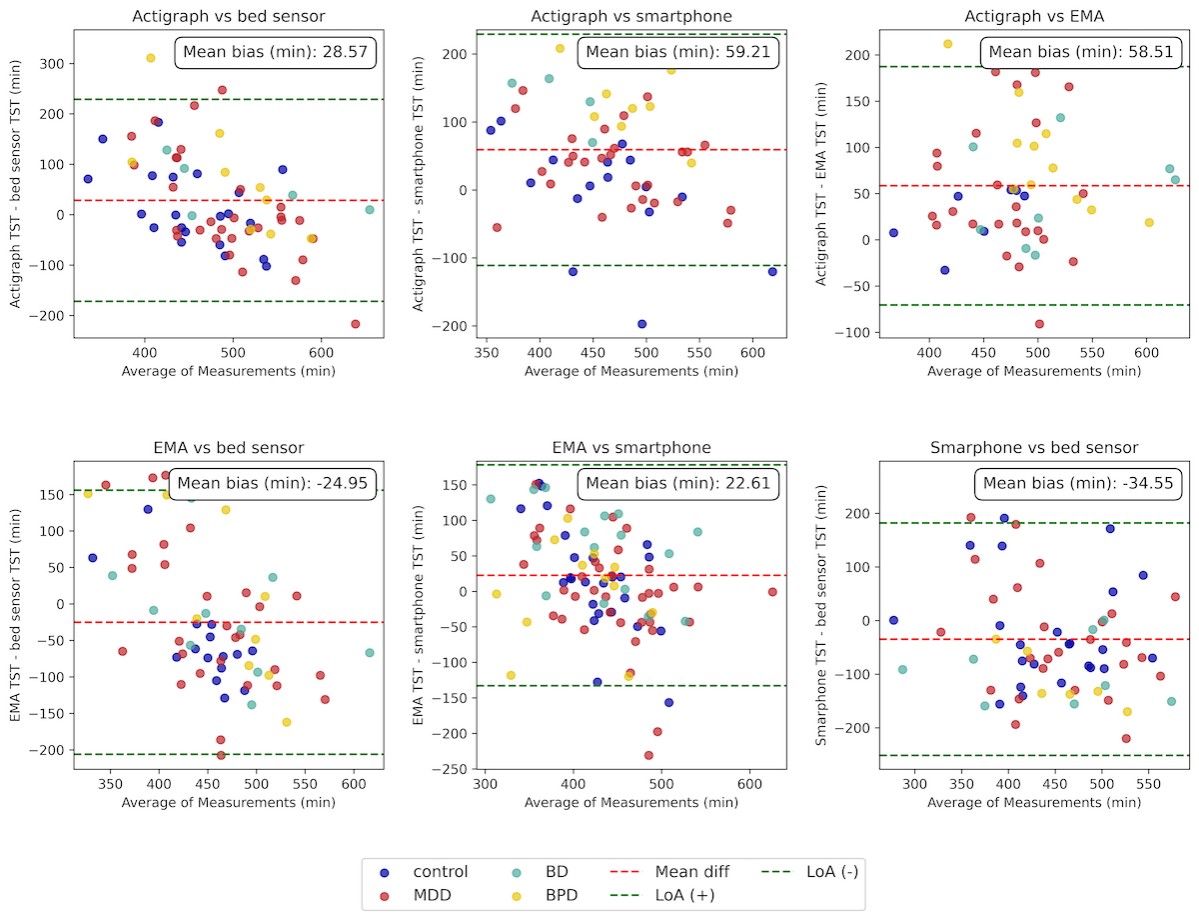
Bland-Altman plot comparing total sleep time (TST) across modalities, with data points color-coded by group. The red dashed line indicates the mean bias, and the green dashed lines represent the 95% limit of agreement (LoA). The actigraph reported higher TST compared to other modalities, while the smartphone reported lower values.

The Bland-Altman plot in Figure 6 showed that the actigraph consistently reported higher TST compared to the other modalities. The mean difference between actigraph and bed sensor in TST was 28.57 (SD 103.03; 95% CI 10.83-46.31) minutes, reflecting a small positive bias, which was not statistically significant (*P*=.13). The actigraph also overestimated TST compared to the smartphone by 59.21 (SD 87.56; 95% CI 44.41-74.00) minutes and by 58.51 (SD 66.42; 95% CI 47.68-69.34) minutes when compared to the EMA data, both of which were statistically significant (*P<.*001).

EMA slightly underestimated TST relative to the bed sensor and actigraph but overestimated it compared to the smartphone. The mean difference between EMA and the bed sensor was –24.95 (SD 93.15; 95% CI –40.08 to –9.82) minutes, reflecting a small negative bias, which was statistically significant (*P*=.01). In comparison to the smartphone, EMA had a mean difference of 22.61 (SD 79.74; 95% CI 9.52-35.69) minutes, indicating a very small positive bias, though this difference was not marginally significant (*P*=.07). Lastly, the smartphone underestimated TST compared to the bed sensor, with a mean difference of –34.55 (SD 111.37; 95% CI –52.82 to –16.26) minutes, which was statistically significant (*P*<*.*001). These results suggest that there are significant biases between the actigraph and both EMA and the smartphone. The actigraph overestimated TST, likely because it can misclassify wakefulness as sleep. EMA, based on subjective recall, slightly underestimated TST. The smartphone showed the greatest underestimation, which may be due to screen activity shortly before sleep onset and after waking.

**Figure 7:**
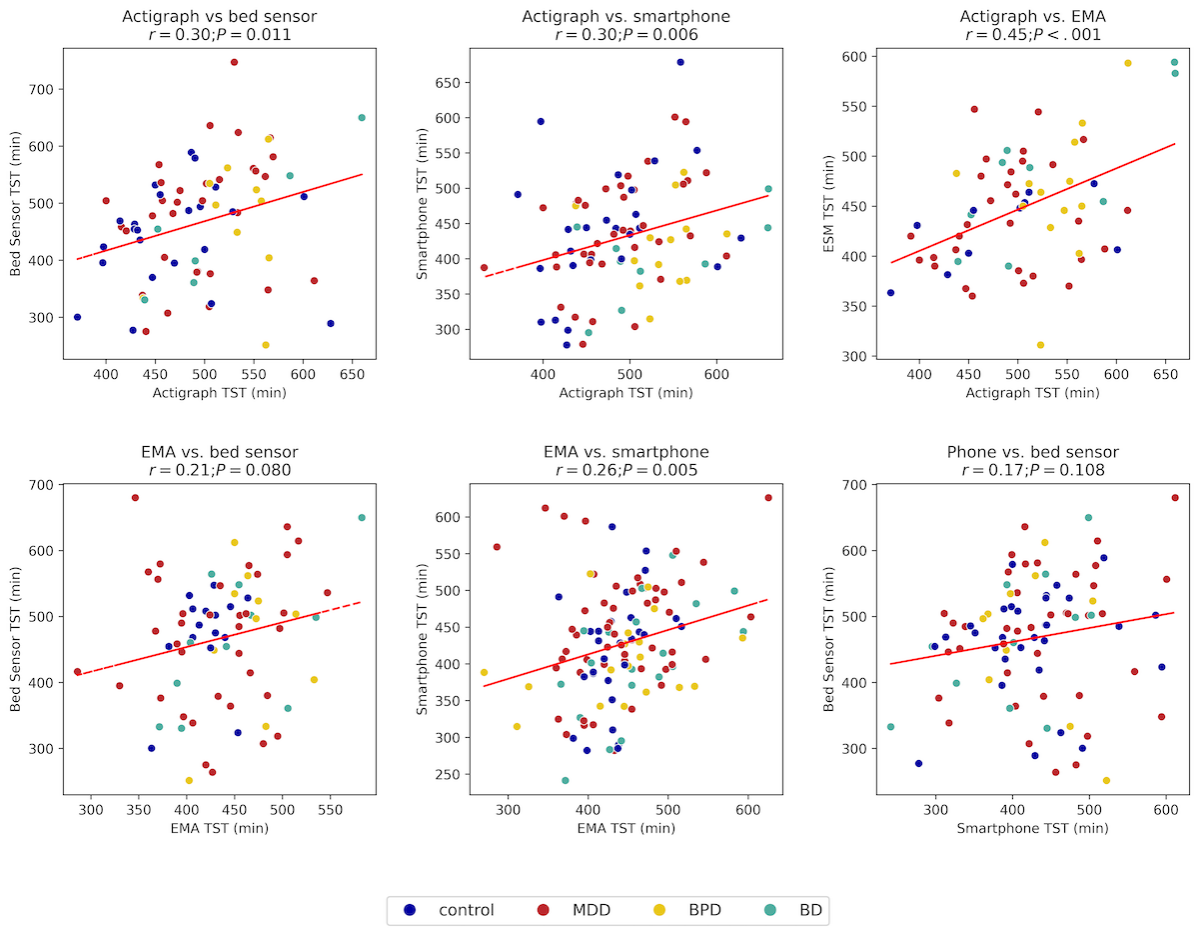
Scatter plots comparing total sleep time (TST) across different modalities. All comparisons show weak positive correlations, with ecological momentary assessment (EMA) versus bed sensor and smartphone versus bed sensor not exhibiting statistically significant correlations.

Pearson correlations revealed varying levels of agreement between modalities for TST (Figure 7). The strongest correlation was observed between actigraph and EMA (*r*=0.45, 95% CI 0.20-0.63; *P*<*.*001), Actigraph and bed sensor sleep measurements demonstrated a low positive correlation (*r*=0.30, 95% CI 0.12-0.91; *P*=*.*01), while actigraph and smartphone measurements showed a similar low correlation (*r*=0.30, 95% CI 0.10-0.60; *P*=*.*006). The correlation between EMA and bed sensor data was less pronounced and marginally significant (*r*=0.21, 95% CI –0.05 to 0.79; *P*=*.*08). EMA and smartphone measurements showed a negligible correlation (*r*=0.26, 95% CI 0.10-0.56; *P*=*.*005). Similarly, smartphone and bed sensor data did not exhibit a significant correlation (*r*=0.17, 95% CI –0.04 to 0.47; *P*=*.*11), suggesting greater variability in smartphone-based sleep estimates.

### Sleep Measurement Discrepancies in Patient and Control Groups

Paired *t* tests (2-tailed) were used to compare sleep onset times between devices and methods for controls and patients separately, with differences reported in minutes. Linear regression with Pearson correlation was also performed to assess the relationship between device pairs.

#### Sleep Onset

Analysis of sleep onset times using paired *t* tests (2-tailed) revealed minimal mean biases (Table 4) between measurement modalities in both control and patient groups. For instance, the mean bias between actigraph and bed sensor was –4.57 minutes in controls (t_22_=–0.37; *P*=.71) and 4.85 minutes in patients (t_47_=0.52; *P*=.60), indicating no systematic difference in onset detection timing. Similarly, the smartphone versus bed sensor comparison showed a mean bias of 18.2 minutes in controls (*P*=.20) but was near zero in patients (0.02 minutes; *P*=.99). Despite these small biases, correlations (Table 5) revealed stronger agreement in patients than controls for some modality pairs. Notably, the actigraph versus bed sensor correlation was 0.74 (*P*<.001) in patients, compared to 0.49 (*P*=.02) in controls, suggesting more consistent relative timing across these modalities in patients. The smartphone versus bed sensor and actigraph versus smartphone onset times also showed significant positive correlations in both groups. These findings suggest that although absolute onset time differences between devices are small and nonsignificant, the consistency between modalities is greater in patient groups.

**Table 4:** Mean bias and statistical comparison of sleep metrics across methods and groups.

Sleep metric, comparison, and group			Mean bias (SD)	*t* test (*df*)	*P* value
**Onset**					
	**Actigraph vs bed sensor**				
		Control	–4.57 (58.9)	–0.37 (22)	.71
		Patients	4.85 (64.2)	0.52 (47)	.60
	**Smartphone vs bed sensor**				
		Control	18.2 (75.2)	1.32 (29)	.20
		Patients	0.02 (91.5)	0.0014 (59)	.99
	**Actigraph vs smartphone**				
		Control	–8.00 (68.2)	–0.59 (24)	.56
		Patients	–2.44 (77.9)	–0.24 (58)	.81
**Offset**					
	**Actigraph vs bed sensor**				
		Control	20.9 (65.8)	1.52 (22)	.14
		Patients	34.9 (93.5)	2.59 (47)	.01
	**Smartphone vs bed sensor**				
		Control	–22.9 (86.0)	–1.46 (29)	.15
		Patients	–45.3 (116.3)	–3.02 (59)	.004
	**Actigraph vs smartphone**				
		Control	32.2 (70.4)	2.29 (24)	.03
		Patients	78.7 (89.9)	6.72 (58)	<.001
**Total sleep time**					
	**Actigraph vs bed sensor**				
		Control	33.9 (105.44)	1.74 (28)	.09
		Patients	28.6 (104.83)	1.10 (69)	.27
	**Actigraph vs EMA**				
		Control	53.3 (61.65)	3.11 (27)	.004
		Patients	61.6 (67.28)	5.10 (69)	<.001
	**Actigraph vs smartphone**				
		Control	8.26 (98.54)	1.69 (28)	.10
		Patients	80.9 (78.04)	6.65 (69)	<.001
	**EMA vs bed sensor**				
		Control	–42.5 (75.975)	–0.70 (27)	.49
		Patients	–20.9 (97.54)	–2.51 (70)	.01
	**EMA vs smartphone**				
		Control	3.65 (80.44)	0.05 (27)	.96
		Patients	18.9 (90.22)	1.65 (92)	.10
	**Smartphone vs bed sensor**				
		Control	–14.5 (100.62)	–0.92 (33)	.36
		Patients	–42.5 (116.72)	–3.57 (70)	<.001

**Table 5:** Correlations across assessment methods between control group and patients.

Sleep metric, comparison, and users				Correlation, *r* (95% CI)	*P* value
**Onset**					
	**Actigraph vs bed sensor**				
		Control	0.49 (0.10 to 0.95)		.02
		Patients	0.74 (0.61 to 1.05)		<.001
	**Smartphone vs bed sensor**				
		Control	0.58 (0.21 to 0.68)		<.001
		Patients	0.48 (0.27 to 0.77)		<.001
	**Actigraph vs smartphone**				
		Control	0.73 (0.59 to 1.38)		<.001
		Patients	0.66 (0.50 to 0.92)		<.001
**Offset**					
	**Actigraph vs bed sensor**				
		Control	0.73 (0.54 to 1.32)		<.001
		Patients	0.59 (0.44 to 1.04)		<.001
	**Smartphone vs bed sensor**				
		Control	0.44 (0.13 to 1.13)		.02
		Patients	0.42 (0.25 to 0.92)		<.001
	**Actigraph vs smartphone**				
		Control	0.46 (0.05 to 0.59)		.02
		Patients	0.49 (0.25 to 0.68)		<.001
**Total sleep time**					
	**Actigraph vs bed sensor**				
		Control	0.12 (–0.30 to 0.55)		.54
		Patients	0.33 (0.06 to 0.61)		.02
	**Actigraph vs EMA**				
		Control	0.55 (–0.02 to 1.13)		.097
		Patients	0.43 (0.18 to 0.68)		.002
	**Actigraph vs smartphone**				
		Control	0.36 (–0.02 to 0.74)		.08
		Patients	0.30 (0.05 to 0.54)		.02
	**EMA vs bed sensor**				
		Control	0.31 (–0.21 to 0.83)		.26
		Patients	0.21 (–0.05 to 0.47)		.12
	**EMA vs smartphone**				
		Control	0.34 (–0.02 to 0.71)		.07
		Patients	0.25 (0.05 to 0.45)		.02
	**Smartphone vs bed sensor**				
		Control	0.17 (0.53 to –0.20)		.38
		Patients	017 (–0.08 to 0.43)		.19

#### Sleep Offset

Significant mean biases were observed across modalities for sleep offset, particularly in the patient group (Table 4). In controls, the mean difference between actigraph and bed sensor was 20.91 minutes but was not significant (*P*=.14). In contrast, this difference increased to 34.94 minutes in patients and was statistically significant (t_47_=2.59; *P*=.01), indicating that patient characteristics such as movement patterns or restlessness affect actigraph readings more strongly. Comparisons involving smartphone also showed larger and significant discrepancies in patients. The smartphone underestimated sleep offset by 45.3 minutes relative to the bed sensor (*P*=.004), whereas in controls the difference was nonsignificant (*P*=.16). The largest offset bias was observed between actigraph and smartphone in patients, with a mean difference of 78.7 minutes (*P*<.001), compared to 32.2 minutes in controls *(P*=.03).

Despite these differences, correlation analyses showed that the relative agreement across modalities remained comparable between groups (Table 5). Actigraph and bed sensor offset times correlated strongly in both controls (*r*=0.73, *P*<.001) and patients (*r*=0.59, *P*<.001). Smartphone and bed sensor correlations were moderate and similar across groups (controls: *r*=0.44; *P*=.02 and patients: *r*=0.42; *P*<.001). Actigraph and smartphone also showed comparable correlations (controls: *r*=0.46; *P*=.02 and patients: *r*=0.49; *P*<.001). These results indicate that absolute disagreement in sleep offset timing was greater among patients, especially involving smartphone data. However, the consistency of measurements across individuals, as reflected by correlation coefficients, was largely preserved.

#### Total Sleep Time

TST comparisons demonstrated the largest mean biases, with notable differences between controls and patients (Table 4). Controls exhibited small, marginally significant biases between modalities, such as 33.9 minutes for actigraph versus bed sensor (*P*=.09) and 8.26 minutes for actigraph versus smartphone (*P*=.10). Conversely, patients showed significantly larger biases: actigraph versus smartphone differed by 80.9 minutes (*P*<.001), actigraph versus EMA by 61.6 minutes (*P*<.001), and actigraph versus bed sensor by 28.6 minutes (*P*=.27). In Table 5, correlations for TST were generally weak and nonsignificant in controls (eg, actigraph vs bed sensor *r*=0.12; *P*=.54), but patients exhibited moderate significant correlations, including actigraph versus bed sensor (*r*=0.33; *P*=.02), actigraph versus smartphone (*r*=0.30; *P*=.02), and actigraph versus EMA (*r*=0.43; *P*=.002). EMA also correlated moderately with smartphone in patients (*r*=0.25; *P*=.02), but not significantly in controls (*r*=0.34; *P*=.07). These findings suggest that while absolute differences in TST between modalities are more pronounced in patients, the modalities capture shared variance in sleep duration better in patient than in control groups.

### Demographic and Seasonal Factors Predicting the Alignments of Sleep Measurement

For each sleep measure, 3 linear mixed-effects models were fitted, using the difference between modalities as the dependent variable and including demographic and seasonal factors as fixed effects. The results are summarized in Tables 6-11.

**Table 6:** Total sleep time (TST) alignment.

Predictors	Actigraph – bed			Smartphone – bed			Actigraph – smartphone	
	Estimate (95% CI)	*P* value	Estimate (95% CI)		*P* value	Estimate (95% CI)		*P* value
Age	0.00 (–0.03 to 0.03)	.97	–0.02 (–0.05 to 0.01)		.11	–0.02 (–0.04 to –0.00)		.048
Sex (female)	–0.61 (–1.51 to 0.29)	.18	–1.03 (–1.74 to –0.33)		.004	0.52 (–0.05 to 1.09)		.08
MEQ^a^	–0.04 (–0.12 to 0.03)	.23	–0.04 (–0.11 to 0.02)		.19	–0.04 (–0.08 to 0.01)		.10
Day length (minutes)	–0.09 (–0.19 to 0.02)	.10	-0.04 (–0.12 to 0.05)		.37	–0.07 (–0.14 to –0.01)		.03
Group: BD^b^	-0.86 (–2.27 to 0.54)	.23	–0.50 (–1.57 to 0.58)		.36	0.89 (0.11 to 1.68)		.03
Group: BPD^c^	0.00 (–1.34 to 1.35)	>99	–0.03 (–1.15 to 1.08)		.95	0.83 (0.01 to 1.64)		.05
Group: MDD^d^	–0.08 (–1.21 to 1.06)	.90	–0.60 (–1.52 to 0.32)		.20	–0.16 (–0.77 to 0.46)		.62

^a^MEQ: Morning-Eveningness Questionnaire.

^b^BD: bipolar disorder.

^c^BPD: borderline personality disorder.

^d^MDD: major depressive disorder.

**Table 7:** Random-effects variance components for total sleep time (TST) alignment.

Metric	Actigraph – bed	Smartphone – bed	Actigraph – smartphone
σ²	2.47	2.84	2.86
τ₀₀ (user)	1.27	0.82	0.74
ICC^a^	0.34	0.22	0.21
Marginal *R*²/Conditional *R*²	0.045/0.369	0.073/0.281	0.111/0.294

^a^ICC: intraclass correlation coefficient.

**Table 8:** Sleep offset alignment.

Predictors	Actigraph – Bed			Smartphone – Bed			Actigraph – Smartphone	
	Estimate (95% CI)	*P* value	Estimate (95% CI)		*P* value	Estimate (95% CI)		*P* value
Age	0.01 (–0.02 to 0.04)	.59	–0.03 (–0.06 to –0.00)		.03	–0.01 (–0.04 to 0.01)		.20
Sex (female)	–0.57 (–1.49 to 0.36)	.23	–0.18 (–0.95 to 0.59)		.64	0.51 (–0.13 to 1.15)		.12
MEQ^a^	–0.05 (–0.12 to 0.03)	.22	0.01 (–0.06 to 0.08)		.68	–0.02 (–0.07 to 0.03)		.46
Daylight duration	–0.11 (–0.22 to –0.00)	.045	–0.11 (–0.20 to –0.01)		.03	–0.07 (–0.14 to 0.00)		.07
Group: BD^b^	–0.33 (–1.78 to 1.11)	.65	–0.32 (–1.47 to 0.83)		.59	0.66 (–0.20 to 1.53)		.13
Group: BPD^c^	0.84 (–0.55 to 2.23)	.24	0.10 (–1.10 to 1.29)		.88	0.83 (–0.07 to 1.74)		.07
Group: MDD^d^	0.67 (–0.50 to 1.84)	.26	–0.13 (–1.12 to 0.86)		.80	–0.15 (–0.84 to 0.53)		.66

^a^MEQ: Morning-Eveningness Questionnaire.

^b^BD: bipolar disorder.

^c^BPD: borderline personality disorder.

^d^MDD: major depressive disorder.

**Table 9:** Random-effects variance components for sleep offset alignment.

Metric	Actigraph – Bed	Smartphone – Bed	Actigraph – Smartphone
σ²	3.15	7.18	7.15
τ₀₀ (user)	1.32	0.67	0.72
ICC^a^	0.30	0.09	0.09
Marginal *R*²/Conditional *R*²	0.075/0.348	0.042/0.124	0.042/0.130

^a^ICC: intraclass correlation coefficient.

**Table 10:** Sleep onset alignment.

Predictors	Actigraph – Bed			Smartphone – BedEstimate			Actigraph – Smartphone	
	Estimate (95% CI)	*P* value	Estimate (95% CI)		*P* value	Estimate (95% CI)		*P* value
Age	0.03 (–0.01 to 0.07)	.20	–0.01 (–0.05 to 0.02)		.56	0.01 (–0.02 to 0.03)		.45
Sex (female)	–0.14 (–1.28 to 1.00)	.81	–0.52 (–1.42 to 0.38)		.26	0.53 (–0.16 to 1.22)		.13
MEQ^a^	–0.01 (–0.10 to 0.08)	.88	–0.04 (–0.12 to 0.05)		.40	–0.04 (–0.09 to 0.02)		.17
Daylight duration	–0.02 (–0.15 to 0.12)	.81	0.00 (–0.10 to 0.11)		.93	–0.03 (–0.11 to 0.04)		.41
Group: BD^b^	–0.40 (–2.18 to 1.39)	.66	–0.19 (–1.57 to 1.19)		.79	0.01 (–0.94 to 0.97)		.98
Group: BPD^c^	0.41 (–1.30 to 2.12)	.64	0.67 (–0.77 to 2.10)		.36	0.69 (–0.29 to 1.67)		.17
Group: MDD^d^	0.68 (–0.75 to 2.12)	.35	–0.25 (–1.42 to 0.93)		.68	0.01 (–0.74 to 0.75)		.99

^a^MEQ: Morning-Eveningness Questionnaire.

^b^BD: bipolar disorder.

^c^BPD: borderline personality disorder.

^d^MDD: major depressive disorder.

**Table 11:** Random-effects variance components for sleep onset alignment.

Metric	Actigraph – Bed	Smartphone – Bed	Actigraph – Smartphone
σ²	2.97	2.47	2.97
τ₀₀ (user)	2.12	1.54	1.16
ICC^a^	0.42	0.38	0.28
Marginal *R*²/Conditional *R*²	0.036/0.437	0.045/0.413	0.045/0.313

^a^ICC: intraclass correlation coefficient.

Older age was associated with higher alignment in TST between actigraphy and the bed sensor (β=−0.02, 95% CI −0.04 to 0.00; *P*=.048), as well as higher alignment in sleep offset between the smartphone and the bed sensor (β=−0.03, 95% CI −0.06 to 0.00; *P*=.03). Males exhibited higher alignment compared with females in TST between smartphone and bed sensor measurements (β=−1.03, 95% CI −1.74 to 0.33; *P*=.004). Longer daylight duration consistently predicted higher alignment in sleep offset for both actigraphy–bed sensor (β=–0.11, 95% CI –0.22 to 0.00; *P*=.045) and smartphone–bed sensor comparisons (β=–0.11, 95% CI −0.20 to −0.01; *P*=.03), highlighting the impact of weather on the alignment between measurement modalities. Patients diagnosed with BD (β=0.89, 95% CI 0.11-1.68; *P*=.03) and BPD (β=0.83, 95% CI 0.01-1.64; *P*=.047) demonstrated lower alignment in TST between actigraphy and bed sensor compared to controls. No significant predictors of sleep onset alignment were identified.

## Discussion

### Principal Findings

Our results showed that patients exhibited greater variability in sleep onset, offset, and TST compared to healthy controls. While actigraphy generally reported longer TST, smartphones typically reported lower values. Sleep onset and offset showed stronger correlations across modalities, whereas TST correlations were weaker. The largest measurement biases were observed for sleep offset timing and TST in patients, with the greatest discrepancies involving smartphone-derived data. However, correlation analyses suggest that relative agreement across devices remains intact within both groups. Further analysis with linear mixed models revealed that older individuals showed better alignment between modalities, for example in TST between actigraphy and the bed sensor and in sleep offset between the smartphone and the bed sensor. In contrast, females and patients with BD or BPD exhibited poorer alignment in TST between modalities. Additionally, longer daylight duration was linked to better alignment in both sleep offset and TST across all modalities.

### Comparison With Previous Work

Accurate sleep monitoring outside of laboratory settings is increasingly relevant for mental health research, particularly in the absence of PSG as ground truth [3]. As evidenced in this study, sleep measurements among individuals with depressive disorders exhibited greater between-individual variability compared to the control group, as reflected by the wider CIs. Patients with depression may experience either insomnia, such as trouble falling asleep, frequent awakenings, or early morning awakenings, or hypersomnia. Hypersomnia is often linked to atypical depression, a subtype of major depressive episodes identified in the *DSM-5* (*Diagnostic and Statistical Manual of Mental Disorders* [Fifth Edition]) [56], and contributes significantly to the variation in sleep patterns among individuals with depression. These findings align with prior research, including Ho et al [34], which reported notable disturbances in both day and night activities among individuals experiencing MDD or symptoms of depression. Their meta-analysis review highlighted disruptions in sleep onset and sleep continuity, poorer sleep efficiency, and greater variability in circadian sleep-wake patterns in relation to healthy controls.

Our results reveal systematic biases in sleep estimation in all measurement modalities. The higher values of TST as measured by actigraphy may be attributed to its limited ability to detect wakefulness during sleep, leading to an overestimation of TST compared to other modalities, a finding consistent with previous studies that have shown similar patterns of overestimation by actigraphy [13,57]. On the other hand, smartphone data may have limitations in accurately capturing sleep onset and offset times. The Bland-Altman plots showed that the differences in sleep onset between the smartphone and other modalities were significantly larger, which could contribute to the smartphone’s underestimation of TST. This aligns with findings from Ciman and Wac [58], who noted that smartphone algorithms, while more accurate in estimating TST, struggle to detect precise sleep onset and offset. They noted that individuals often stay in bed after waking up before using their smartphones, making it difficult for the smartphone to detect the precise offset time. Similarly, smartphone use before sleep may delay the detected sleep onset. Moreover, the smartphone’s underestimation of TST in this study is consistent with previous research, such as Natale et al [59], which found that smartphone generally underestimated TST in healthy individuals, particularly during longer TSTs.

Our findings indicate that smartphone-based measurements exhibited the lowest mean bias and a positive correlation of TST with EMA data. These results partly align with those of Maynard et al [60], who found that a smartphone app strongly correlated with self-reported sleep diaries. However, while their study reported that the smartphone overestimated TST compared to actigraphy in healthy adults, our results indicated that actigraphy consistently recorded higher TST, with healthy controls showing a small, marginally significant bias (8.26 minutes; *P*=.10) and patients exhibiting a much larger, significant bias (80.89 minutes; *P*<.001). Some discrepancy may stem from methodological differences, as Maynard’s study used a proprietary algorithm to convert accelerometer data into sleep metrics, while this study relied on a simpler approach based on smartphone screen activity events tracking. Given the limited number of studies comparing smartphone-based sleep measurements, Maynard’s study represents the closest methodological comparison. This further highlights the novelty of this study and enhances the validity of our results by showing that smartphone-based sleep measurements can provide meaningful insights even in clinical populations.

Regarding psychiatric populations, Staples et al [61] conducted one of the few studies to compare smartphone-based sleep monitoring in individuals with schizophrenia. They found that smartphone accelerometer estimates of TST were moderately correlated with self-reported TST (*r*=0.69). However, their study did not compare smartphone estimates directly with actigraphy or other modalities, making it difficult to fully interpret their findings. This study, which directly compared smartphone-derived TST estimates with those from other objective modalities in psychiatric populations, contributes to the sparse literature on this topic [20]. We emphasize the necessity for additional research to gain a deeper understanding of the accuracy of smartphone sleep monitoring in clinical populations, particularly when compared to other objective modalities.

Furthermore, the weak correlation in TST across different methods suggests that each device captures distinct aspects of sleep, leading to measurement inconsistencies. Such direct comparisons across multiple modalities within patient groups, without using PSG as a ground truth, are rarely conducted, making this study a unique contribution. One of the few studies in this area is Piantino et al [62], which identified a strong relationship between a bed sensor and an actigraphy device for metrics such as bed entry time, sleep onset, sleep offset, bed exit time, and TST in healthy individuals. However, Piantino et al also acknowledged that the consistency of the two modalities could be reduced in specific groups, such as individuals with depression or sleep disorders, who may be more likely to lie motionless in bed without achieving sleep. Additionally, in this study, some psychiatric patients were recruited from psychiatric units rather than primary care, where individuals often present with more severe symptoms. As a result, they may be more likely to be overmedicated. Sedative medications can also have muscle-relaxing effects, which may contribute to this phenomenon.

### Factors Associated With the Alignments of Sleep Measurements

Demographic factors such as age and gender were associated with sleep measurement alignment. Older individuals exhibited better alignment between smartphone and actigraphy estimates, possibly because of more stable sleep patterns. Another explanation could be the reduced smartphone use before bedtime by older participants [63], which may align with actigraphy’s overestimation tendencies. This also aligns with findings from Jonasdottir et al [64], who analyzed a large global dataset of over 11 million nights from wearable devices across 47 countries. Their study confirmed that older adults exhibit shorter TST, earlier sleep timing, and lower intraindividual variability compared with younger adults, which could contribute to more consistent sleep measurement alignment. Gender differences also played a role, as women showed more discrepancies between smartphone and bed sensor measurements. Jonasdottir et al [64] also found that women experience more frequent nighttime awakenings than men, particularly in early to middle adulthood. This suggests that increased sleep fragmentation in women may lead to greater inconsistencies between sensor-based measurements.

Additionally, day length appeared to correlate with better alignment, likely because extended daylight promotes more consistent sleep-wake schedules. These findings align with prior research suggesting that external cues, such as light exposure, play a significant role in regulating sleep timing [30-34]. This is particularly important given that the study was conducted in Finland, a country prone to extreme fluctuations in daylight. Such fluctuations may have a substantial impact on sleep patterns, making it critical to study and track these changes closely to better understand their effects on sleep and circadian rhythms.

Lastly, the greater alignment observed in patients with BD and those with BPD patients reinforces the established link between psychiatric disorders and sleep disturbances. Our findings also emphasize the comorbidity between these conditions, consistent with previous literature [65,66]. This is also supported by research linking these conditions to irregular sleep patterns. For instance, a meta-analysis found significant variations between patients with BPD and healthy participants in sleep stability and architecture indicators, including sleep efficiency, sleep onset delay, TST, and rapid eye movement sleep parameters [12]. Similarly, studies examining BD have highlighted disruptions in both quantity and quality, which are often influenced by the manic and depressive episodes characteristic of the disorder [67].

When using sleep monitoring to assess clinical status or detect early changes, it is important to recognize that there is no definitive standard for directly comparing different modalities. However, we can compare TST with subjective reports from EMA. Although no single device can be definitively identified as the best, we can identify which devices align closely with subjective reports. Our findings showed that, in terms of bias, smartphone had the lowest bias compared to EMA, although the difference was not statistically significant and may be due to random measurement error. Following this, the bed sensor exhibited the next lowest bias, while the actigraph had the highest bias. In contrast, when considering correlation, the actigraph and EMA showed the strongest alignment, suggesting that actigraphy may show a more consistent correlation with subjective sleep reports. However, because actigraphy has been shown to overestimate TST, it is possible that individuals also tend to overestimate their own TST. We note that additional context may contribute to device misalignment. For instance, co-sleeping can distort heart rate signals and the sleep metrics derived from them. Conversely, habitual snoozers usually experience brief awakenings to turn off an alarm, thus underestimating smartphone-based sleep estimates. Clinicians should take these nuances into account, along with the various factors that influence agreement between devices, when choosing a method for sleep monitoring.

### Limitations and Future Work

A key strength of this study is its naturalistic approach, allowing for real-world sleep monitoring without interfering with participants’ routines. This enhances ecological validity and provides insights into sleep measurement in everyday settings, particularly for individuals with depressive disorders. Additionally, using multiple modalities allows for a comprehensive comparison of different methodologies, highlighting their relative strengths and weaknesses. However, there are important limitations to consider. First, this study did not include PSG or sleep logs, as these could have disrupted participants’ natural sleep patterns. Incorporating PSG would have posed significant challenges due to the need for frequent laboratory visits, which could have increased participant burden and potentially led to higher dropout rates, especially among patients. While this approach improves feasibility, it also means that we lack a clinical gold standard for validation. Without PSG data, determining the absolute accuracy of each device remains challenging. Second, although EMA provides subjective estimates of TST that align with some objective modalities, it remains susceptible to sleep misperception, where individuals may misjudge their sleep quality or duration [22]. Third, this study includes a relatively small sample size and short follow-up period, which may impact the generalizability of the results. Additionally, the sleep/wake labels used in the study were generated by proprietary algorithms provided by the manufacturers, which may limit the accuracy and comparability of the sleep data. Moreover, differences in sleep patterns may be partly attributable to the use of sleep medication, which is common among psychiatric patients but not typically used by healthy participants. TST outside the 3-13 hour range were excluded based on standard guidelines, which are suitable for healthy individuals but may overlook cases of hypersomnia in patients with depression who may sleep more than 13 hours. Finally, the study was conducted in a single location, resulting in a lack of population diversity. These limitations should be considered when interpreting the results, particularly in clinical contexts.

Future research could include replication of the study in countries with different climates or daylight patterns to determine whether places with different seasonal patterns also influence sleep characteristics in individuals. Studies incorporating PSG or validated sleep logs could help improve measurement accuracy while exploring ways to minimize participant burden. Larger and more diverse samples, as well as longer follow up periods, would allow for stronger conclusions regarding the stability of sleep patterns over time. Future work could also compare proprietary device algorithms with standardized scoring methods to evaluate the consistency of measurements. Examining sleep patterns among participants who use or do not use sleep medication may help clarify how medication influences digital sleep measures. In addition, more detailed clinical symptom data should be collected, as the present analyses lacked detailed information on specific sleep symptoms, such as insomnia or hypersomnia. Using validated measures to distinguish these sleep disturbances would improve understanding of how particular sleep problems relate to different sleep modalities and rest–activity patterns.

### Conclusions

This study highlights the feasibility of using actigraphy, smartphone data, and bed sensors for sleep tracking in naturalistic settings for patients with depressive disorders. However, it also reveals systematic biases in sleep offset across these modalities, which contribute to discrepancies in TST. Furthermore, our findings highlight that factors such as age, sex, clinical diagnosis, and daylight duration can influence the alignment of sleep measurements across different modalities. This is particularly important in psychiatric populations, where accurate and reliable sleep tracking is essential for research and clinical care.

## Abbreviations

BD: bipolar disorder
BPD: borderline personality disorder
DSM-5: Diagnostic and Statistical Manual of Mental Disorders (Fifth Edition)
EMA: ecological momentary assessment
LoA: limit of agreement
MDD: major depressive disorder
MEQ: Morning-Eveningness Questionnaire
PSG: polysomnography
TST: total sleep time

## Data Availability

The datasets generated or analyzed during this study are not publicly available due to containing highly sensitive personal information and restrictions in the research permit, but are available from the corresponding author on reasonable request for purposes of scientific collaboration.
